# 2-(2-Methyl­anilino)-*N*′-(propan-2-yl­idene)acetohydrazide

**DOI:** 10.1107/S1600536809044353

**Published:** 2009-10-31

**Authors:** Muhammad Salim, Zaid Mahmood, M. Nawaz Tahir, Saeed Ahmad, Azhar Saeed

**Affiliations:** a Institute of Chemistry, University of the Punjab, Lahore, Pakistan; b Department of Physics, University of Sargodha, Sargodha, Pakistan; c Department of Chemistry, Gomal University, Dera Ismail Khan, Pakistan

## Abstract

The conformation of the title compound, C_12_H_17_N_3_O, is consolidated by an intra­molecular N—H⋯O hydrogen bond, generating an *S*(5) ring. In the crystal, inversion dimers linked by pairs of N—H⋯O inter­actions occur, resulting in *R*_2_^2^(8) ring motifs.

## Related literature

For related structures, see: Salim *et al.* (2009 ▶); Shi *et al.* (2007 ▶). For graph-set theory, see: Bernstein *et al.* (1995 ▶).
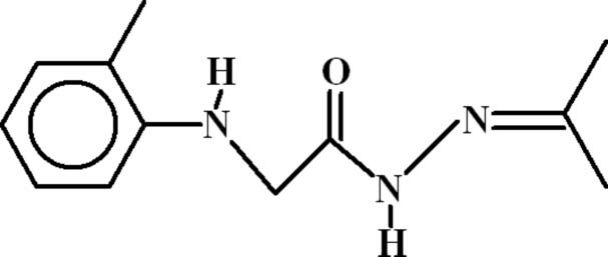


## Experimental

### 

#### Crystal data

C_12_H_17_N_3_O
*M* *_r_* = 219.29
Monoclinic, 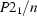

*a* = 13.2194 (9) Å
*b* = 4.3865 (3) Å
*c* = 21.7413 (13) Å
β = 103.433 (3)°
*V* = 1226.22 (14) Å^3^
*Z* = 4
Mo *K*α radiation
μ = 0.08 mm^−1^
*T* = 296 K
0.28 × 0.25 × 0.22 mm

#### Data collection

Bruker Kappa APEXII CCD diffractometer
Absorption correction: multi-scan (*SADABS*; Bruker, 2005 ▶) *T*_min_ = 0.979, *T*_max_ = 0.984
13485 measured reflections
2998 independent reflections
1608 reflections with *I* > 2σ(*I*)
*R*_int_ = 0.040

#### Refinement

*R*[*F* ^2^ > 2σ(*F* ^2^)] = 0.048
*wR*(*F* ^2^) = 0.166
*S* = 1.00
2998 reflections
148 parameters
H atoms treated by a mixture of independent and constrained refinement
Δρ_max_ = 0.18 e Å^−3^
Δρ_min_ = −0.16 e Å^−3^

### 

Data collection: *APEX2* (Bruker, 2007 ▶); cell refinement: *SAINT* (Bruker, 2007 ▶); data reduction: *SAINT*; program(s) used to solve structure: *SHELXS97* (Sheldrick, 2008 ▶); program(s) used to refine structure: *SHELXL97* (Sheldrick, 2008 ▶); molecular graphics: *ORTEP-3* (Farrugia, 1997 ▶) and *PLATON* (Spek, 2009 ▶); software used to prepare material for publication: *WinGX* (Farrugia, 1999 ▶) and *PLATON*.

## Supplementary Material

Crystal structure: contains datablocks global, I. DOI: 10.1107/S1600536809044353/hb5183sup1.cif

Structure factors: contains datablocks I. DOI: 10.1107/S1600536809044353/hb5183Isup2.hkl

Additional supplementary materials:  crystallographic information; 3D view; checkCIF report

## Figures and Tables

**Table 1:** Hydrogen-bond geometry (Å, °)

*D*—H⋯*A*	*D*—H	H⋯*A*	*D*⋯*A*	*D*—H⋯*A*
N1—H1⋯O1	0.81 (3)	2.21 (3)	2.604 (2)	110 (2)
N2—H2⋯O1^i^	0.88 (2)	2.06 (2)	2.920 (2)	166 (2)

Symmetry code: (i) 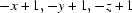.

**Fig. 1.**
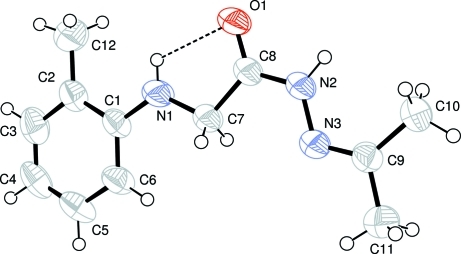
View of (I) with displacement ellipsoids drawn at the 50% probability level. The dotted line represent the intramolecular H-bond.

**Fig. 2.**
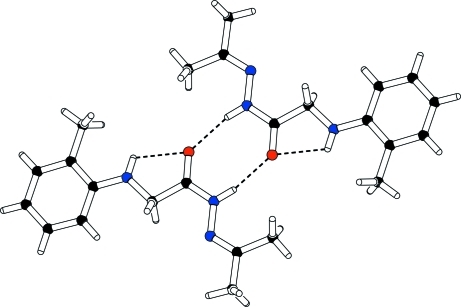
The partial packing of (I), which shows that molecules form inversion dimers due to H-bondings.

## supplementary crystallographic information

### Comment

Literature has shown that hydrazides as well as their derivatives are characterized by low toxicity and possess a broad spectrum of pharmaceutical activities. The title compound (I, Fig. 1) is one of the several hydrazide derivatives prepared with substitution and alteration of the basic moiety as a key to obtain good potency. In this context we have already reported the crystal structure of 2-(3,4-Dimethylanilino)acetohydrazide (Salim *et al.*, 2009).

The crystal structure of (II) 2-(1*H*-Benzotriazol-1-yl)-*N*'-(propan-2-ylidene)acetohydrazide (Shi *et al.*, 2007) has been published which contains the side chain of (I).

In (I) the 2-methylanilinic group A (C1—C6/N1/C12) and the side chain group B (C7/C8/N2/N3/C9—C11/O1) are planar with maximum r. m. s. deviations of 0.0064 and 0.0146 Å respectively, from the respective mean square planes. The dihedral angle between A/B is 4.70 (10)°. In (I), there exists intramolecular H-bonding of N—H···O type (Table 1, Fig. 1) completing S(5) ring motif (Bernstein *et al.*, 1995). The molecules are dimerized due to intermolecular H-bonding of N—H···O type (Table 1, Fig. 2) completing *R*_2_^2^(8) ring motif. There does not exist any C–H···π or π···π interactions.

### Experimental

2-[(2-Methylphenyl)amino]acetohydrazide (0.9 g, 5 mmol) and acetone (0.29 g, 5 mmol) were refluxed along with stirring in 100 ml of ethylalcohol for 30 minutes and after evaporation of the solvent, the crude product obtained was recrystallized in methylalcohol to obtain colorless prisms of (I).

### Refinement

The coordinates of H1, H2, H7A and H7B were refined. The other H-atoms were positioned geometrically (C–H = 0.93–0.96 Å) and refined as riding with *U*_iso_(H) = 1.2*U*_eq_(carrier) or 1.5*U*_eq_(methyl C).

### Crystal data

**Table:**

C_12_H_17_N_3_O	*F*(000) = 472
*M**_r_* = 219.29	*D*_x_ = 1.188 Mg m^−^^3^
Monoclinic, *P*2_1_/*n*	Mo *K*α radiation, λ = 0.71073 Å
Hall symbol: -P 2yn	Cell parameters from 2998 reflections
*a* = 13.2194 (9) Å	θ = 3.0–28.2°
*b* = 4.3865 (3) Å	µ = 0.08 mm^−^^1^
*c* = 21.7413 (13) Å	*T* = 296 K
β = 103.433 (3)°	Prism, colourless
*V* = 1226.22 (14) Å^3^	0.28 × 0.25 × 0.22 mm
*Z* = 4	

### Data collection

**Table:**

Bruker Kappa APEXII CCD diffractometer	2998 independent reflections
Radiation source: fine-focus sealed tube	1608 reflections with *I* > 2σ(*I*)
graphite	*R*_int_ = 0.040
Detector resolution: 7.40 pixels mm^-1^	θ_max_ = 28.2°, θ_min_ = 3.0°
ω scans	*h* = −17→17
Absorption correction: multi-scan (*SADABS*; Bruker, 2005)	*k* = −5→5
*T*_min_ = 0.979, *T*_max_ = 0.984	*l* = −28→28
13485 measured reflections	

### Refinement

**Table:**

Refinement on *F*^2^	Primary atom site location: structure-invariant direct methods
Least-squares matrix: full	Secondary atom site location: difference Fourier map
*R*[*F*^2^ > 2σ(*F*^2^)] = 0.048	Hydrogen site location: inferred from neighbouring sites
*wR*(*F*^2^) = 0.166	H atoms treated by a mixture of independent and constrained refinement
*S* = 1.00	*w* = 1/[σ^2^(*F*_o_^2^) + (0.067*P*)^2^ + 0.3661*P*] where *P* = (*F*_o_^2^ + 2*F*_c_^2^)/3
2998 reflections	(Δ/σ)_max_ < 0.001
148 parameters	Δρ_max_ = 0.18 e Å^−^^3^
0 restraints	Δρ_min_ = −0.16 e Å^−^^3^

### Special details

**Table:**

Geometry. Bond distances, angles *etc*. have been calculated using the rounded fractional coordinates. All su's are estimated from the variances of the (full) variance-covariance matrix. The cell e.s.d.'s are taken into account in the estimation of distances, angles and torsion angles
Refinement. Refinement of *F*^2^ against ALL reflections. The weighted *R*-factor *wR* and goodness of fit *S* are based on *F*^2^, conventional *R*-factors *R* are based on *F*, with *F* set to zero for negative *F*^2^. The threshold expression of *F*^2^ > σ(*F*^2^) is used only for calculating *R*-factors(gt) *etc*. and is not relevant to the choice of reflections for refinement. *R*-factors based on *F*^2^ are statistically about twice as large as those based on *F*, and *R*- factors based on ALL data will be even larger.

### Fractional atomic coordinates and isotropic or equivalent isotropic displacement parameters (Å^2^)

**Table:**

	*x*	*y*	*z*	*U*_iso_*/*U*_eq_	
O1	0.47742 (12)	0.3771 (4)	0.42354 (7)	0.0780 (6)	
N1	0.53042 (16)	0.2794 (5)	0.31712 (8)	0.0739 (7)	
N2	0.61215 (13)	0.6771 (4)	0.46619 (8)	0.0572 (6)	
N3	0.70384 (12)	0.8122 (4)	0.46023 (7)	0.0550 (5)	
C1	0.54130 (15)	0.2216 (5)	0.25657 (8)	0.0537 (6)	
C2	0.46554 (16)	0.0463 (5)	0.21591 (9)	0.0597 (7)	
C3	0.47980 (19)	−0.0121 (6)	0.15595 (10)	0.0751 (9)	
C4	0.5629 (2)	0.0969 (6)	0.13542 (10)	0.0768 (9)	
C5	0.63565 (18)	0.2686 (6)	0.17511 (10)	0.0704 (8)	
C6	0.62626 (15)	0.3307 (5)	0.23575 (9)	0.0622 (7)	
C7	0.59948 (16)	0.4699 (5)	0.36058 (9)	0.0551 (7)	
C8	0.55802 (15)	0.5040 (5)	0.41921 (8)	0.0536 (6)	
C9	0.75244 (15)	0.9750 (4)	0.50608 (9)	0.0524 (6)	
C10	0.71970 (17)	1.0357 (6)	0.56608 (9)	0.0674 (8)	
C11	0.85192 (17)	1.1136 (6)	0.49869 (11)	0.0728 (8)	
C12	0.3731 (2)	−0.0733 (7)	0.23729 (13)	0.0865 (10)	
H1	0.478 (2)	0.222 (6)	0.3270 (12)	0.0887*	
H2	0.5864 (16)	0.694 (5)	0.4998 (11)	0.0686*	
H3	0.43077	−0.13040	0.12853	0.0902*	
H4	0.56980	0.05417	0.09469	0.0922*	
H5	0.69216	0.34476	0.16126	0.0845*	
H6	0.67688	0.44581	0.26275	0.0747*	
H7A	0.6053 (16)	0.673 (5)	0.3444 (10)	0.0661*	
H7B	0.6679 (17)	0.383 (5)	0.3735 (10)	0.0661*	
H10A	0.65304	1.13419	0.55670	0.1011*	
H10B	0.76993	1.16536	0.59273	0.1011*	
H10C	0.71507	0.84643	0.58744	0.1011*	
H11A	0.86472	1.05450	0.45867	0.1092*	
H11B	0.90797	1.04383	0.53216	0.1092*	
H11C	0.84711	1.33164	0.50050	0.1092*	
H12A	0.39624	−0.20103	0.27369	0.1299*	
H12B	0.33007	−0.18975	0.20383	0.1299*	
H12C	0.33383	0.09441	0.24803	0.1299*	

### Atomic displacement parameters (Å^2^)

**Table:**

	*U*^11^	*U*^22^	*U*^33^	*U*^12^	*U*^13^	*U*^23^
O1	0.0743 (10)	0.1126 (13)	0.0574 (9)	−0.0338 (9)	0.0366 (7)	−0.0246 (8)
N1	0.0746 (12)	0.1060 (16)	0.0500 (10)	−0.0339 (11)	0.0325 (9)	−0.0234 (10)
N2	0.0590 (10)	0.0746 (12)	0.0433 (9)	−0.0111 (8)	0.0229 (7)	−0.0086 (8)
N3	0.0568 (9)	0.0646 (10)	0.0474 (9)	−0.0067 (8)	0.0196 (7)	−0.0032 (8)
C1	0.0570 (11)	0.0650 (12)	0.0420 (10)	0.0042 (9)	0.0173 (8)	−0.0040 (9)
C2	0.0613 (12)	0.0670 (13)	0.0512 (11)	0.0067 (10)	0.0141 (9)	−0.0076 (10)
C3	0.0838 (16)	0.0852 (17)	0.0534 (12)	0.0070 (13)	0.0099 (11)	−0.0179 (12)
C4	0.0926 (17)	0.0973 (18)	0.0456 (11)	0.0234 (15)	0.0262 (12)	−0.0069 (12)
C5	0.0678 (13)	0.0986 (18)	0.0523 (12)	0.0206 (13)	0.0291 (11)	0.0085 (12)
C6	0.0561 (11)	0.0851 (15)	0.0489 (11)	0.0012 (10)	0.0192 (9)	−0.0021 (10)
C7	0.0580 (11)	0.0679 (14)	0.0431 (10)	−0.0073 (10)	0.0195 (9)	−0.0039 (10)
C8	0.0571 (11)	0.0649 (12)	0.0426 (10)	−0.0054 (10)	0.0192 (8)	−0.0049 (9)
C9	0.0565 (10)	0.0556 (11)	0.0467 (10)	−0.0007 (9)	0.0152 (8)	0.0010 (9)
C10	0.0738 (14)	0.0787 (15)	0.0522 (12)	−0.0106 (12)	0.0198 (10)	−0.0122 (11)
C11	0.0697 (13)	0.0839 (16)	0.0685 (14)	−0.0171 (12)	0.0234 (11)	−0.0067 (12)
C12	0.0758 (15)	0.102 (2)	0.0831 (17)	−0.0268 (14)	0.0212 (13)	−0.0220 (15)

### Geometric parameters (Å, °)

**Table:**

O1—C8	1.225 (3)	C9—C10	1.491 (3)
N1—C1	1.381 (2)	C3—H3	0.9300
N1—C7	1.423 (3)	C4—H4	0.9300
N2—N3	1.382 (2)	C5—H5	0.9300
N2—C8	1.339 (3)	C6—H6	0.9300
N3—C9	1.272 (2)	C7—H7A	0.97 (2)
N1—H1	0.81 (3)	C7—H7B	0.96 (2)
N2—H2	0.88 (2)	C10—H10A	0.9600
C1—C2	1.402 (3)	C10—H10B	0.9600
C1—C6	1.390 (3)	C10—H10C	0.9600
C2—C3	1.384 (3)	C11—H11A	0.9600
C2—C12	1.500 (4)	C11—H11B	0.9600
C3—C4	1.365 (4)	C11—H11C	0.9600
C4—C5	1.361 (3)	C12—H12A	0.9600
C5—C6	1.380 (3)	C12—H12B	0.9600
C7—C8	1.507 (3)	C12—H12C	0.9600
C9—C11	1.491 (3)		
			
C1—N1—C7	123.25 (19)	C4—C5—H5	120.00
N3—N2—C8	119.78 (16)	C6—C5—H5	120.00
N2—N3—C9	117.48 (16)	C1—C6—H6	120.00
C7—N1—H1	117.5 (18)	C5—C6—H6	120.00
C1—N1—H1	118.7 (18)	N1—C7—H7A	113.1 (13)
N3—N2—H2	124.1 (15)	N1—C7—H7B	112.1 (13)
C8—N2—H2	116.2 (15)	C8—C7—H7A	106.9 (13)
N1—C1—C2	118.99 (19)	C8—C7—H7B	107.1 (13)
N1—C1—C6	121.23 (19)	H7A—C7—H7B	108.8 (19)
C2—C1—C6	119.78 (17)	C9—C10—H10A	109.00
C3—C2—C12	121.8 (2)	C9—C10—H10B	109.00
C1—C2—C3	117.5 (2)	C9—C10—H10C	109.00
C1—C2—C12	120.72 (19)	H10A—C10—H10B	109.00
C2—C3—C4	122.7 (2)	H10A—C10—H10C	109.00
C3—C4—C5	119.4 (2)	H10B—C10—H10C	109.00
C4—C5—C6	120.5 (2)	C9—C11—H11A	109.00
C1—C6—C5	120.14 (19)	C9—C11—H11B	109.00
N1—C7—C8	108.46 (18)	C9—C11—H11C	109.00
O1—C8—N2	121.29 (17)	H11A—C11—H11B	109.00
O1—C8—C7	120.90 (18)	H11A—C11—H11C	109.00
N2—C8—C7	117.81 (18)	H11B—C11—H11C	109.00
N3—C9—C11	116.23 (18)	C2—C12—H12A	109.00
C10—C9—C11	117.65 (18)	C2—C12—H12B	109.00
N3—C9—C10	126.12 (19)	C2—C12—H12C	109.00
C2—C3—H3	119.00	H12A—C12—H12B	109.00
C4—C3—H3	119.00	H12A—C12—H12C	109.00
C3—C4—H4	120.00	H12B—C12—H12C	109.00
C5—C4—H4	120.00		
			
C7—N1—C1—C2	−176.0 (2)	C6—C1—C2—C12	−179.9 (2)
C7—N1—C1—C6	4.8 (3)	N1—C1—C6—C5	179.7 (2)
C1—N1—C7—C8	174.7 (2)	C2—C1—C6—C5	0.5 (3)
C8—N2—N3—C9	179.89 (19)	C1—C2—C3—C4	−0.9 (4)
N3—N2—C8—O1	178.75 (19)	C12—C2—C3—C4	179.4 (2)
N3—N2—C8—C7	−1.3 (3)	C2—C3—C4—C5	0.4 (4)
N2—N3—C9—C10	−0.2 (3)	C3—C4—C5—C6	0.5 (4)
N2—N3—C9—C11	179.05 (18)	C4—C5—C6—C1	−0.9 (4)
N1—C1—C2—C3	−178.8 (2)	N1—C7—C8—O1	0.2 (3)
N1—C1—C2—C12	0.9 (3)	N1—C7—C8—N2	−179.80 (19)
C6—C1—C2—C3	0.4 (3)		

### Hydrogen-bond geometry (Å, °)

**Table:**

*D*—H···*A*	*D*—H	H···*A*	*D*···*A*	*D*—H···*A*
N1—H1···O1	0.81 (3)	2.21 (3)	2.604 (2)	110 (2)
N2—H2···O1^i^	0.88 (2)	2.06 (2)	2.920 (2)	166 (2)

Symmetry codes: (i) −*x*+1, −*y*+1, −*z*+1.
